# ﻿*Hotwheels* gen. nov., a new ground spider genus (Araneae, Gnaphosidae) from southwest China

**DOI:** 10.3897/zookeys.1189.115996

**Published:** 2024-01-18

**Authors:** Bo Liu, Feng Zhang

**Affiliations:** 1 The Key Laboratory of Zoological Systematics and Application, Hebei Basic Science Center for Biotic Interaction, College of Life Sciences, Hebei University, Baoding, Hebei 071002, China Hebei University Baoding China

**Keywords:** Description, morphology, new species, taxonomy

## Abstract

A new monotypic ground spider genus, *Hotwheels***gen. nov.**, is described, with the type species *H.sisyphus***sp. nov.** (♂♀) from southwest China. This new genus is not assigned to any of the known subfamilies of Gnaphosidae, belonging only to the *Echemus* group of genera. It resembles *Synaphosus* Platnick & Shadab, but it can be differentiated by the presence of a median apophysis. Descriptions, illustrations and a records map are provided.

## ﻿Introduction

Gnaphosidae is a highly diverse spider family, containing 2466 extant species in 151 genera (WSC 2023). Southwest China mostly lies within the Oriental Realm and has the highest diversity of spiders in China (Holt et al. 2013; Yao and Li 2021), including 87 species of 25 genera of gnaphosid spiders (WSC 2023). Three new ground spider genera (*Meizhelan* Lin & Li, 2023, *Platnickus* Liu & Zhang, 2023, *Yuqilin* Lin & Li, 2023) have been reported from southwest China in just 2023 (Lin and Li 2023; Liu and Zhang 2023). However, ground spider research in southwest China and neighbouring countries is severely limited, with a lack of regional revisions (WSC 2023). Our knowledge of the diversity of gnaphosids in southwest China is far from adequate, and many new taxa remain to be discovered.

While examining the ground spider collections from southwest China, we found several specimens of an unknown species resembling *Synaphosus* Platnick & Shadab, 1980 in somatic characters and genitalic structures, but it can be differentiated by the presence of a median apophysis, which indicates a new genus should be established.

## ﻿Material and methods

All specimens were preserved in 75% ethanol and examined and measured under a Leica M205A stereomicroscope. Photographs were taken using an Olympus BX51 microscope equipped with a Kuy Nice CCD camera and were imported into Helicon Focus v.7 for stacking. Final figures were retouched using Adobe Photoshop 2020. A map was generated in QGIS v.3.24.2. All measurements are given in millimeters. Leg measurements are shown as: total length (femur, patella, tibia, metatarsus, tarsus). Epigynes were removed and cleared in a pancreatin solution (Álvarez-Padilla and Hormiga 2007). All specimens studied are deposited in the Museum of Hebei University (**MHBU**), Baoding, China.

Morphological terminology follows Azevedo et al. (2017). Abbreviations used in this study are: ALE, anterior lateral eye; AME, anterior median eye; BH, basal haematodocha; C, conductor; CA, apophysis of conductor; CD, copulatory duct; CO, copulatory opening; DSS, duct of secondary spermatheca; DTM, distal tubular membrane; E, embolus; ED, ejaculatory duct; EP, embolar process; FD, fertilization duct; H, hood; MA, median apophysis; MaAm, major ampullate gland spigots; MH, median haematodocha; Pi, piriform gland spigots; PLE, posterior lateral eye; PME, posterior median eye; PS, primary spermatheca; R, embolar radix; RTA, retrolateral tibial apophysis; SD, sperm duct; SS, secondary spermathecae; ST, subtegulum; T, tegulum; TM, terminal membrane of embolus.

## ﻿Taxonomy

### ﻿Family Gnaphosidae Banks, 1892

#### ﻿Genus Hotwheels gen. nov.

Taxon classificationAnimaliaAraneaeGnaphosidae

47A66C3D-F1B2-55FE-A8E4-A34D01E838A4

https://zoobank.org/1E5BA5C9-E641-4F3D-B4DC-ED0525F6416E

##### Type species.

*Hotwheelssisyphus* sp. nov.

##### Etymology.

The generic name refers to Hot Wheels, a collectible die-cast toy car made by Mattel, as the long, coiled embolus of this new genus resembles a Hot Wheels track; neuter in gender.

##### Diagnosis.

The new genus resembles *Synaphosus* Platnick & Shadab, 1980 by metatarsi III and IV having a preening brush, the male palp has a long embolus and large conductor, and the epigyne has a hood and long copulatory ducts (Figs 2–4C, D, 5). It can be distinguished from *Synaphosus* by: 1) the presence of a median apophysis (Figs 2, 3A–D) vs. median apophysis absent (Fig. 3F, G; Ovtsharenko et al. 1994: figs 12–14); 2) the basal half of the embolus which rotates counterclockwise (Fig. 2A) vs. basal half of the embolus rotates clockwise (Ovtsharenko et al. 1994: figs 12–14; Marusik and Omelko 2018: figs 8–11, 20–24, 26–36); 3) a weakly sclerotized conductor without an apophysis or outgrowth (Figs 2A, 3A–D) vs. a partially sclerotized conductor with an apophysis or outgrowth (Fig. 3F, G; Marusik and Omelko 2018: figs 8–11, 20–24, 26–36); and 4) the copulatory duct is circular, wide anteriorly, and almost twice the width of the primary spermathecae (Fig. 5) vs. copulatory duct twisted, narrow anteriorly, and narrower than the primary spermathecae (Ovtsharenko et al. 1994: figs 15, 16; Marusik and Omelko 2018: figs 5–7, 12, 13, 17–19, 39–41).

##### Description.

**Figure 1.**
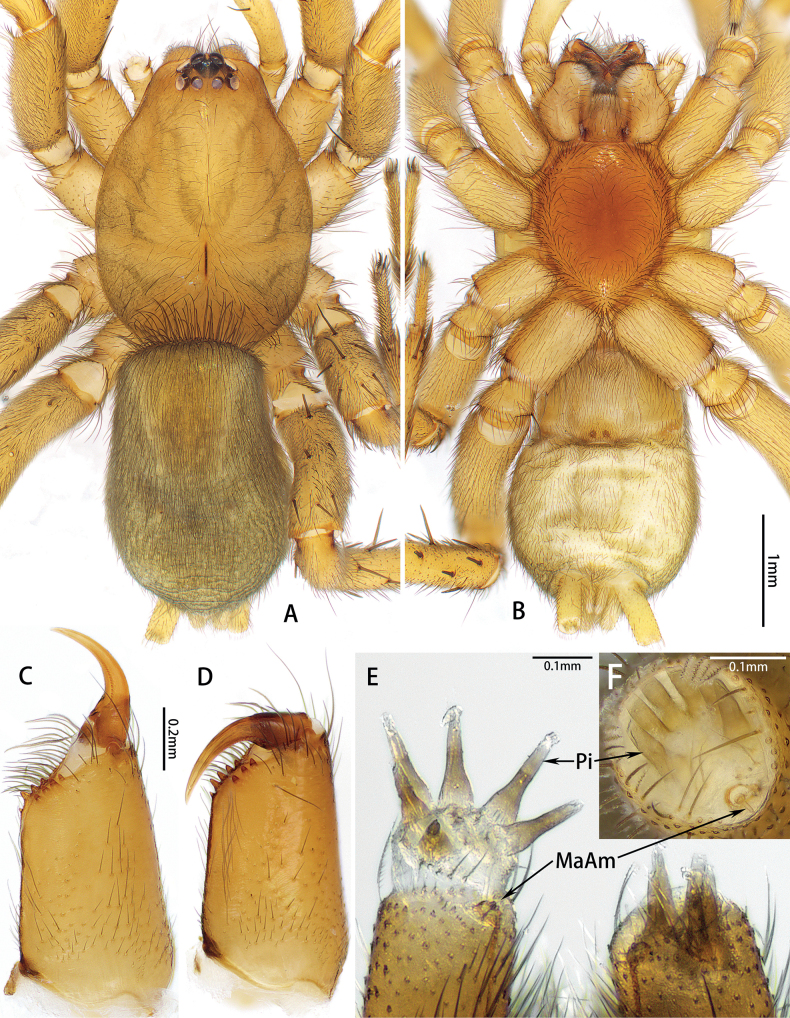
*Hotwheelssisyphus* sp. nov., male (**A–C, F**) and female (**D, E**) **A, B** habitus, dorsal and ventral view **C, D** left chelicera, retrolateral view **E, F** spigots on anterior lateral spinneret. Abbreviations: MaAm = major ampullate gland spigot, Pi = piriform gland spigot.

Small-sized (total length: males = 4.86–5.44; females = 5.45–5.98). In dorsal view, carapace elongate-ovoid, anterior eye row slightly recurved, posterior eye row straight; PME oblique, flat (Figs 1A, 4A). Cheliceral promargin with 4 or 5 teeth, retromargin with 3 or 4 teeth (Fig. 1C, D). Leg formula: 4123. Trochanters not notched. Metatarsi III and IV with preening brushes. Sternum elongate oval, with straight anterior edge, pointed posteriorly (Figs 1B, 4B). Anterior lateral spinnerets with 6 enlarged piriform gland spigots, separated by almost 1.2 times their diameter (Fig. 1E, F). Color in alcohol (Figs 1A, B, 4A, B): carapace yellow-brown; cephalic groove and radial furrow black; fovea distinct, longitudinal. Legs yellow-brown. Abdomen grey, males with anterior dorsal scutum, almost half of abdominal length and more than half of width.

##### Male palp and epigyne.

Same as for the species.

##### Comments.

Murphy (2007) made a formal grouping of gnaphosids without subfamilies, Azevedo et al. (2017) based subfamilies on the results of a morphological phylogenetic analysis, and Lin and Li (2020) erected a monotypic subfamily, Solitudinae. *Hotwheels* sp. nov. cannot be placed in any known Gnaphosidae subfamily because the cheliceral promargin has 4 or 5 teeth, and the retromargin has 3 or 4 teeth (Fig. 1C, D) vs. cheliceral promargin with keel in Herpyllinae (Azevedo et al. 2017: fig. 20e, g) and cheliceral retromargin with a serrated keel or a rounded lamina in Gnaphosinae (Azevedo et al. 2017: fig. 20b, d, f); metatarsi III and IV with preening brush (Fig. 4C, D) vs. metatarsi III and IV with preening comb in Zelotinae (Azevedo et al. 2017: fig. 22h); fertilization ducts directed laterally (Fig. 5B, D) vs. fertilization ducts directed posteriorly in Leptodrassinae (Ott 2012: fig. 40); trochanters not notched (Fig. 1B) vs. trochanters notched in Drassodinae (Azevedo et al. 2017: fig. 24); leg IV tarsus straight (Fig. 4C, D) vs. leg IV tarsus curved in Solitudinae (Lin and Li 2020: fig. 1E, F). It can be placed in the *Echemus* group of genera by the abdomens plain-coloured dorsally and males having an anterior dorsal scutum (Figs 1A, B, 4A, B) (Murphy 2007).

##### Composition.

Only the type species.

#### ﻿ Hotwheels sisyphus sp. nov.

Taxon classificationAnimaliaAraneaeGnaphosidae

5FF7B714-F497-5E4D-BE12-57E8423F7BA5

https://zoobank.org/0437E3C2-DC00-4BA4-865A-E6E6DDDB55A0

**Figure 2.**
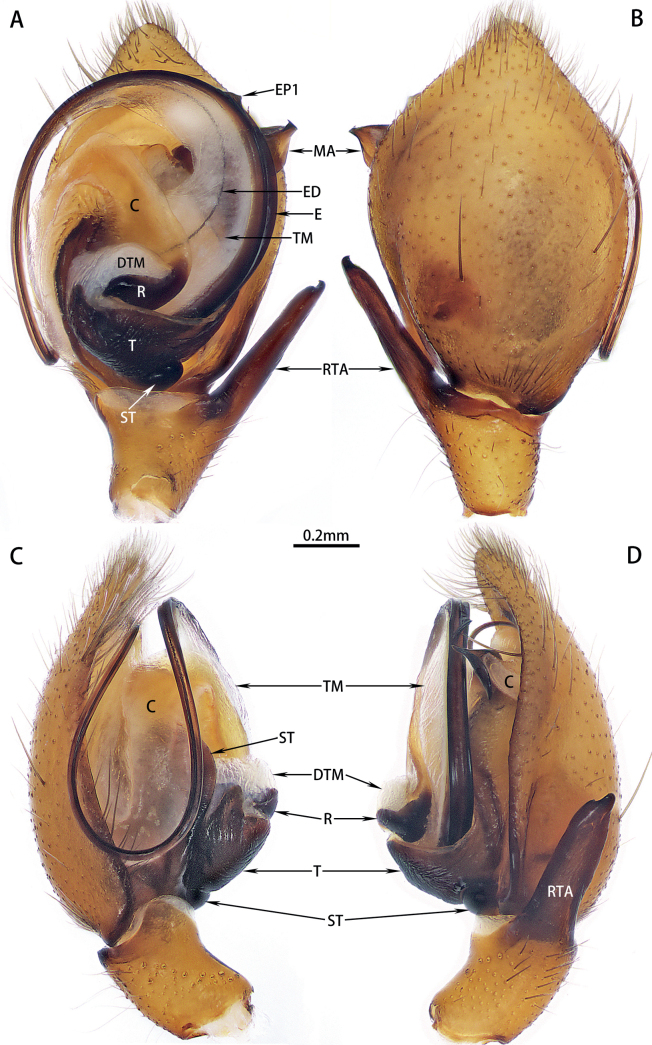
Male left palp of *Hotwheelssisyphus* sp. nov. **A** ventral view **B** dorsal view **C** prolateral view **D** retrolateral view. Abbreviations: C = conductor, DTM = distal tubular membrane, E = embolus, ED = ejaculatory duct, EP1 = embolar process, MA = median apophysis, R = embolar radix, RTA= retrolateral tibial apophysis, ST = subtegulum, T = tegulum, TM = terminal membrane.

**Figure 3.**
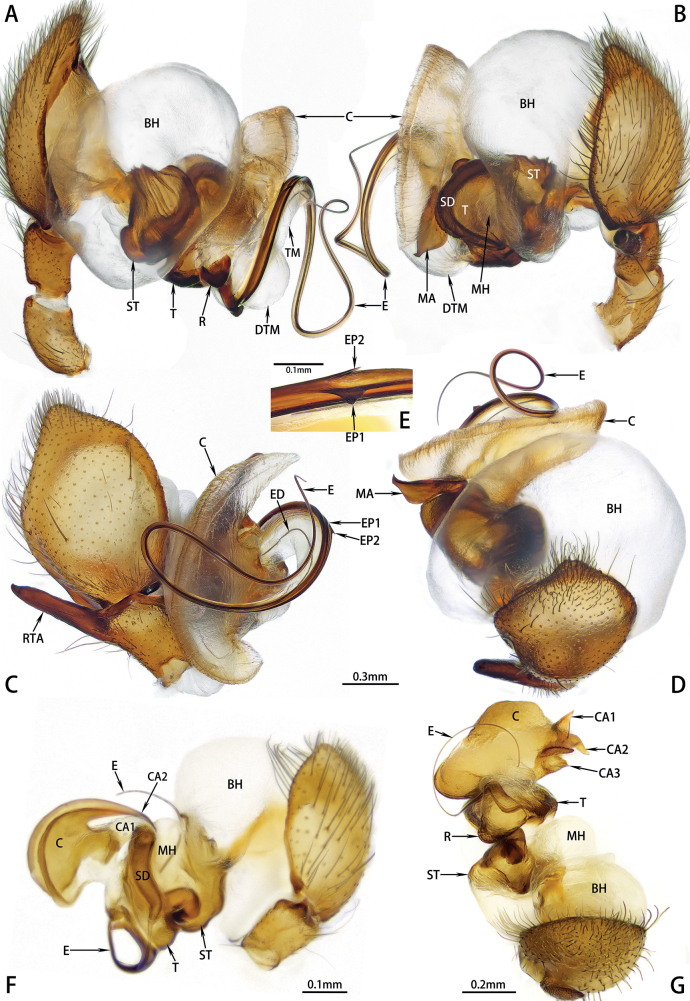
Expanded male left palp of *Hotwheelssisyphus* sp. nov. (**A–E**), *Synaphosuspalearcticus* Ovtsharenko, Levy & Platnick, 1994 (**F**) and *S.cangshanus* Yang, Yang & Zhang, 2013 (**G**). **A** prolateral view **B** retrolateral view **C** dorsal view **D** frontal view **E** embolar processes **F** retrolateral view **G** anterior view. Abbreviations: C = conductor, CA1–3 = apophysis of conductor, BH = basal haematodocha, DTM = distal tubular membrane, E = embolus, ED = ejaculatory duct, EP1–2 = embolar process, MA = median apophysis, MH = median haematodocha, R = embolar radix, RTA= retrolateral tibial apophysis, SD = sperm duct, ST = subtegulum, T = tegulum, TM = terminal membrane.

**Figure 4.**
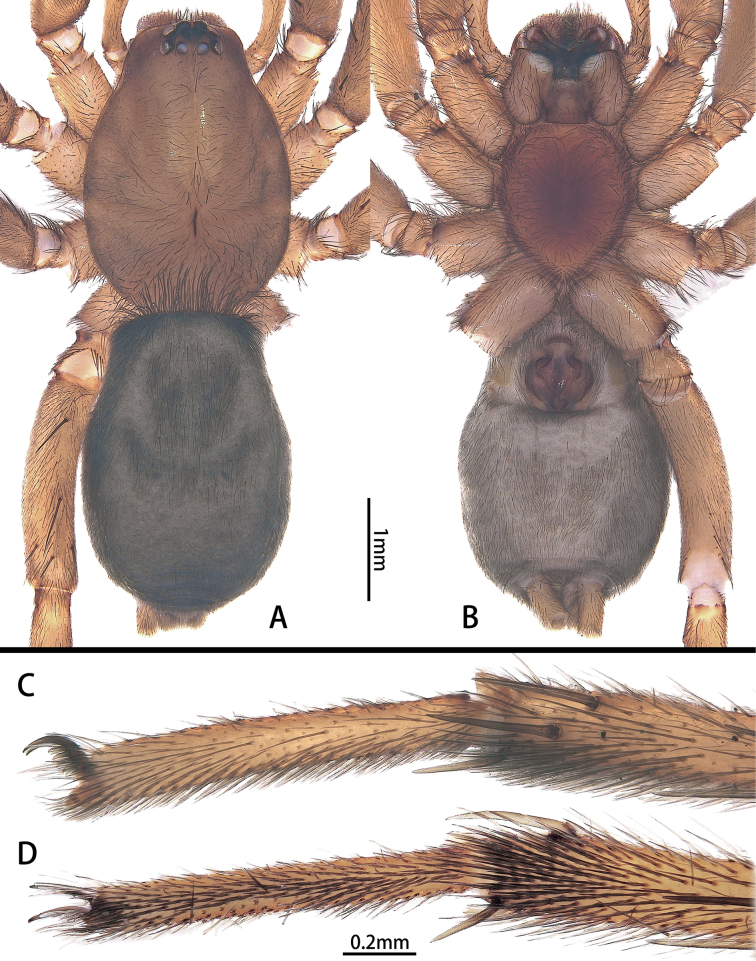
*Hotwheelssisyphus* sp. nov., female **A, B** habitus, dorsal and ventral view **C, D** right tarsus and metatarsus IV, preening brush, in prolateral (**C**) and ventral view (**D**).

**Figure 5.**
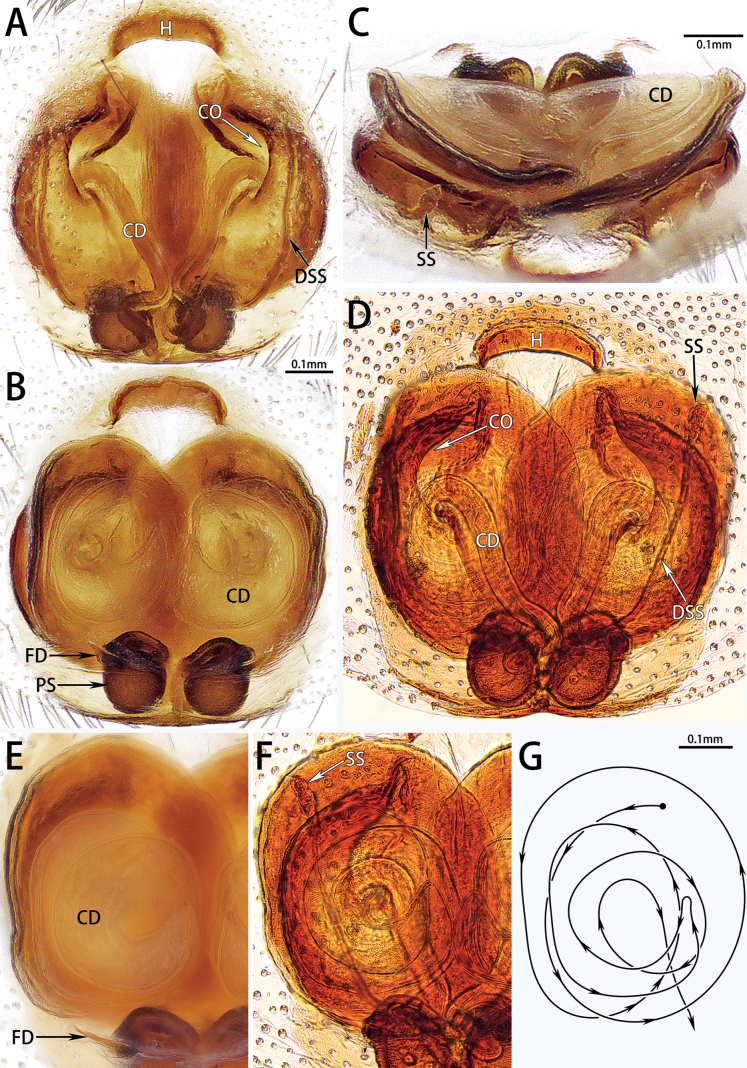
Epigyne of *Hotwheelssisyphus* sp. nov., intact (**A–C, E, G**) or macerated in clove oil (**D, F**), in ventral (**A, D**), frontal (**C**) and dorsal (**B, E, F**) view **G**CD path. Abbreviations: CD = copulatory duct, CO = copulatory opening, DSS = duct of secondary spermatheca, FD = fertilization duct, H = hood, PS = primary spermatheca, SS = secondary spermatheca.

Figs 1 , 2 , 3 , 4 , 5 西西弗斯火轮蛛

##### Type material.

***Holotype*** ♂, China: Guizhou Prov., Bijie City, Hezhang Co., Yemachuan Town, Dayan Cave, 27.132997°N, 104.818279°E, 1392 m elev., 2.X. 2019, leg. Z. Feng & L. Zhao. ***Paratype***: 1♀1♂, same data as holotype; 1♂1♀, China: Guizhou Prov., Qianxinan Buyei and Miao Autonomous Pref., Xingren City, Xinlongchang Town, Lianzhuang Vil., Daxiao Cave, 25.438033°N, 105.116197°E, 1473 m elev., 5.VIII.2022, leg. Y. Hou & L. Zhang; 1♂, China: Sichuan Prov., Leshan City, Emei Mt, Jiulinggang, 29.558433°N, 103.347167°E, 1811 m elev., 13.IV.2018, leg. Z. Zhang & L. Wang; 1♂1♀, China: Yunnan Prov., Honghe Autonomous Pref., Mile Co., Hongxi Town, Bailong Cave, 1.IV.2018, leg. H. Wang.

##### Etymology.

The specific name is derived from Sisyphus, a king in Greek mythology who offended Zeus and whose punishment was to repeatedly roll a huge stone up a hill only to have it roll back down, because the circular copulatory ducts are like Sisyphus’s cyclic mission; noun in apposition.

##### Description.

**Male. *Holotype*** (Fig. 1A, B): total length 5.08; carapace 2.56 long, 1.95 wide; abdomen 2.52 long, 1.70 wide. ***Eye sizes and interdistances***: AME 0.15, ALE 0.16, PME 0.10, PLE 0.13; AME–AME 0.04, AME–ALE 0.01, PME–PME 0.08, PME–PLE 0.02, ALE–PLE 0.03. ***Leg measurements***: I 7.28 (2.11, 0.92, 1.76, 1.39, 1.10), II 6.18 (1.83, 0.80, 1.48, 1.13, 0.94), III 5.62 (1.62, 0.65, 1.18, 1.26, 0.91), IV 7.89 (1.97, 0.81, 1.91, 2.06, 1.14). Cheliceral promargin and retromargin with 4 teeth (Fig. 1C).

***Palp*** in regular state (Fig. 2). Femur and patella unmodified. Tibia with long retrolateral apophysis, nearly 2× longer than tibia, with prolateral curved tip. Cymbium pear shaped, without apical spines. Median apophysis on retrolateral apex of tegulum, nearly 2× wider than tegulum, pointed, curved. Conductor weakly sclerotized, folded and covered on tegulum and subtegulum, posterior part hidden behind median apophysis. Distal tubular membrane connects radix to tegulum. Embolus long, originates at about 7–8 o’clock, basal half rotated anticlockwise, with terminal membrane and two embolar processes (EP1, EP2), posterior half usually hidden behind conductor. Ejaculatory duct distinct.

Expanded palp (Fig. 3A–E). Basal haematodocha large, well developed. Subtegulum smaller than tegulum. Median haematodocha small. Conductor originates at tegulum prolaterally, expanded, crescent shaped with thickened border. Distal tubular membrane expanded, spherical. Terminal membrane inflated.

**Female. *Paratype*** (Fig. 4): total length 5.77; carapace 2.83 long, 2.02 wide; abdomen 2.94 long, 2.05 wide. ***Eye sizes and interdistances***: AME 0.16, ALE 0.15, PME 0.11, PLE 0.13; AME–AME 0.03, AME–ALE 0.01, PME–PME 0.09, PME–PLE 0.06, ALE–PLE 0.04. ***Leg measurements***: I 7.15 (2.16, 0.98, 1.68, 1.22, 1.11), II 6.17 (1.84, 0.90, 1.39, 1.12, 0.92), III 5.98 (1.62, 0.67, 1.24, 1.38, 1.07), IV 8.34 (2.37, 0.80, 1.80, 2.25, 1.12). Cheliceral promargin with 5 teeth, retromargin with 3 teeth (Fig. 1D).

***Epigyne*** (Fig. 5). Epigynal plate elongated oval. Anterior folds form hood. Copulatory openings large, distinct, located mediolaterally. Copulatory ducts long, wide anteriorly, almost twice as wide as primary spermathecae, circular anteriorly and medially, membranous medially. Primary spermathecae small and globular. Secondary spermathecae small, with long ducts. Fertilization ducts extend laterally.

##### Distribution.

**Figure 6.**
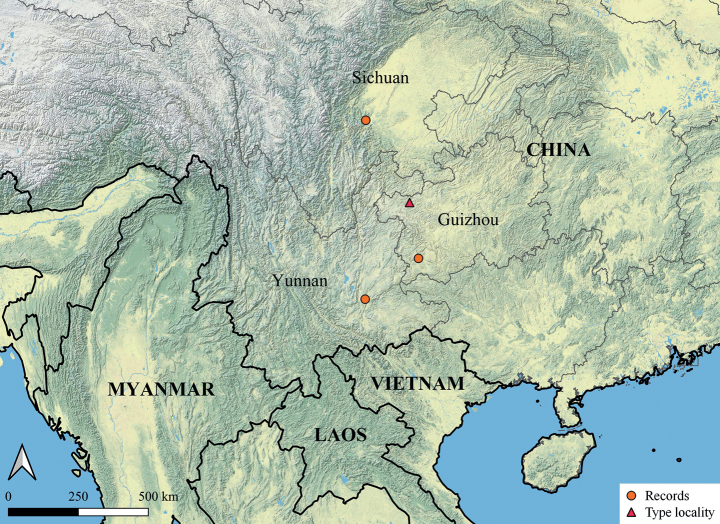
Map showing type locality and other records of *Hotwheelssisyphus* sp. nov.

China (Guizhou, Sichuan, Yunnan) (Fig. 6).

## Supplementary Material

XML Treatment for Hotwheels

XML Treatment for Hotwheels sisyphus

## ﻿Additional information

### Author ORCIDs

Bo Liu https://orcid.org/0000-0003-3740-7173

Feng Zhang https://orcid.org/0000-0002-3347-1031

### Data availability

All of the data that support the findings of this study are available in the main text.

## References

[B1] Álvarez-Padilla F Hormiga G (2007) A protocol for digesting internal soft tissues and mounting spiders for scanning electron microscopy. The Journal of Arachnology 35(3): 538–542. 10.1636/Sh06-55.1

[B2] Azevedo GHF Griswold CE Santos AJ (2017) Systematics and evolution of ground spiders revisited (Araneae, Dionycha, Gnaphosidae). Cladistics 34(6): 579–626. 10.1111/cla.12226 34706482

[B3] Holt BG Lessard JP Borregaard MK Fritz SA Araújo MB Dimitrov D Fabre PH Graham CH Graves GR Jønsson KA Nogués-Bravo D Wang Z Whittaker RJ Fjeldså J Rahbek C (2013) An update of Wallace’s zoogeographic regions of the world. Science 339(6115): 74–78. 10.1126/science.1228282 23258408

[B4] Lin YJ Li SQ (2020) Description on *Solitudesdushengi* gen. nov et sp. nov. from Xinjiang, China (Araneae: Gnaphosidae). Zoological Systematics 45: 312–315. 10.11865/zs.202036

[B5] Lin YJ Li SQ (2023) On nine ground spiders from Xishuangbanna, China (Araneae, Gnaphosidae), including two new genera and seven new species. ZooKeys 1174: 141–174. 10.3897/zookeys.1174.106340 38313332 PMC10838569

[B6] Liu B Zhang F (2023) Revision of the genus *Scopoides* Platnick, 1989 from China, with description of a new genus (Araneae, Gnaphosidae). ZooKeys 1172: 203–215. 10.3897/zookeys.1172.105034 37547180 PMC10398562

[B7] Marusik YM Omelko MM (2018) New data on *Synaphosus* (Araneae: Gnaphosidae) from Southeast Asia. Zootaxa 4374(2): 235–248. 10.11646/zootaxa.4374.2.4 29689798

[B8] Murphy J (2007) Gnaphosid Genera of the World. British Arachnological Society, Cambridgeshire, 46–47.

[B9] Ott R (2012) *Neodrassex*, a new genus of the *Leptodrassex* group (Araneae, Gnaphosidae) from South America. Iheringia. Série Zoologia 102(3): 343–350. 10.1590/S0073-47212012000300015

[B10] Ovtsharenko VI Levy G Platnick NI (1994) A review of the ground spider genus *Synaphosus* (Araneae, Gnaphosidae). American Museum Novitates 3095: 1–27.

[B11] WSC (2023) World Spider Catalog. Version 24. Natural History Museum Bern. 10.24436/2 [Accessed on 1 December 2023]

[B12] Yao ZY Li SQ (2021) Annual report of Chinese spider taxonomy in 2020. Shengwu Duoyangxing 29(8): 1058–1063. 10.17520/biods.2021140

